# Artificial Intelligence in Breast Imaging: Opportunities, Challenges, and Legal–Ethical Considerations

**DOI:** 10.5152/eurasianjmed.2023.23360

**Published:** 2023-12-01

**Authors:** Irmak Durur-Subasi, Ş. Barış Özçelik

**Affiliations:** 1 Istanbul Medipol University International Faculty of Medicine, Istanbul, Turkey; 2 Bilkent University Faculty of Law, Ankara, Turkey

**Keywords:** Artificial Intelligence, Breast, Deep Learning, Imaging, Medical Law

## Abstract

This review explores the transformative impact of artificial intelligence (AI) in breast imaging, driven by a global rise in breast cancer cases. Propelled by deep learning techniques, AI shows promise in refining diagnostic processes, yet adoption rates vary. Its ability to manage extensive datasets and process multidimensional information holds potential for advancing precision medicine in breast cancer research. However, integration faces challenges, from data-related obstacles to ensuring transparency and trust in decision-making. Legal considerations, including the formation of AI teams and intellectual property protection, influence health care’s adoption of AI. Ethical dimensions underscore the need for responsible AI implementation, emphasizing autonomy, well-being, safety, transparency, and accessibility. Establishing a robust legal and ethical framework is crucial for conscientiously deploying AI, ensuring positive impacts on patient safety and treatment efficacy. As nations and organizations aspire to engage in global competition, not merely as consumers, the review highlights the critical importance of developing legal regulations. A comprehensive approach, from AI team formation to end-user processes, is essential for navigating the complex terrain of AI applications in breast imaging. Legal experts play a key role in ensuring compliance, managing risks, and fostering resilient integration. The ultimate goal is a harmonious synergy between technological advancements and ethical considerations, ushering in enhanced breast cancer diagnostics through responsible AI utilization.

**Main Points**

- Specific statistics for Turkey indicate a significant rise in breast cancer cases from 1998 to 2018.
- Mammography is the primary screening method that conclusively reduces breast cancer mortality.
- Breast radiology is a labor-intensive and intricate field, requiring strict standardization and meticulous attention across various stages.
- Artificial intelligence has the potential to help by forecasting breast cancer risk, categorizing lesions, exploring radiogenomics, and predicting responses to treatment.
- The legal experts have important roles in navigating the landscape, ensuring compliance, and highlighting the adherence to ethical principles, including transparency, accountability, and inclusivity, for the responsible integration of AI technologies in health care.

## Introduction

By 2020, breast cancer have surpassed lung and prostate cancers as the most common cancers diagnosed worldwide. Globally, the prevalence of breast cancer is rising, and in the United States, 364 000 instances will be predicted by 2040.^1^ According to Globocan and the data from the Cancer Department of the Ministry of Health of the Republic of Turkey, Turkey’s overall incidence rate climbed from 32.3 per 100 000 in 1998 to 60 per 100 000 in 2018.^2,3^

Any breast cancer screening initiative aims to decrease the incidence and death rate associated with breast cancer by detecting small cancers in their early stages, facilitating precise diagnosis and effective treatment. Among the various screening methods, mammography is the only modality that has demonstrated a conclusive reduction in mortality, contributing to the global implementation of screening programs centered around mammography.^4^

Mammography has its limitations and may require additional, complementary, or advanced imaging. Apart from screening, diagnostic imaging studies with mammography, ultrasound, or magnetic resonance imaging are conducted for women with various symptoms, and the breast imaging examination frequently involves multiple imaging modalities, including interventional procedures.^5^

Breast radiology is a labor-intensive and challenging field that requires meticulous attention to multiple steps. The service steps encompass everything from patient transportation to communication (patient–counselor, patient–physician, physician–physician), patient triage (diagnostic evaluation of pregnant or breastfeeding women, breast cancer assessment in patients under 40, assessment of children, evaluation of men), identification of patient priorities, invitation of follow-up patients, in-service training of staff, standardization of imaging quality, calibration of devices, assignment of examinations to physicians, reporting, determination of additional examination needs by the physician, appropriate use of Breast Imaging Reporting and Data System (BI-RADS), comparison with previous examinations, report writing, integration of clinical and laboratory data with imaging findings, control and approval of results, informing patients, integration of findings from different imaging methods, identification of alternative imaging methods, planning of biopsies, modality selection, biopsy results, and radiological–pathological correlation (decision on how and with which method to follow up, repeat biopsy, or decide on surgical biopsy) to personalized applications. This field requires strict standardization in practice to be free from subjectivity and errors.^6-18^

This article endeavors to shed light on the present status of breast cancer, with a specific emphasis on exploring the transformative capabilities of artificial intelligence (AI) applications in breast imaging. It delves into potential challenges and legal considerations associated with this paradigm, offering insights grounded in contemporary knowledge.

## Breast Radiology and Artificial Intelligence

Radiology is an intricate system, from triage to imaging, interpretation, and advanced procedures. Despite extensive training, standardization efforts, accreditations, in-service training, and inspections, the potential for encountering errors persists due to the complexity of numerous service steps. These areas present opportunities for AI to support the system in enhancing, regulating, correcting, and substituting processes. Despite these expectations, identifying the obstacles to the development and utilization of AI may be the most crucial step for any country or organization that aims to participate in global competition and intends not to remain merely as a customer in the market.^19-20^

The interpretation constitutes a substantial time commitment for physicians involved in breast imaging. Key challenges involve the absence of standard macroscopic anatomy for women’s breasts, the diverse and dense internal structures resulting from their glandular nature, variations in energy and imaging positions across different radiological methods, and limited functional information, particularly in conventional imaging. Additionally, the ongoing need to make decisions for each lesion and finding, interruptions in work due to patient and physician priorities, and the continuous and stressful nature of the work, exacerbated by an increased workload, further contribute to these challenges.^10,11,13,15,21-31^

The exponential rise in demand for imaging and the anticipated decline in the labor to provide reports will put increasing strain on breast imaging. With a growing interest in implementing AI to enhance workflow effectiveness and patient outcomes, solutions to lessen these demands are being looked for.^32^ Artificial intelligence has been employed across various breast imaging techniques, including mammography, ultrasound, and magnetic resonance imaging, in diverse clinical contexts. Radiomics and AI research are advancing swiftly, presenting numerous potential applications in breast imaging, including forecasting breast cancer risk, identifying and categorizing lesions, exploring radiogenomics, and predicting responses to treatment and clinical outcomes.^33^

**Figure 1.**
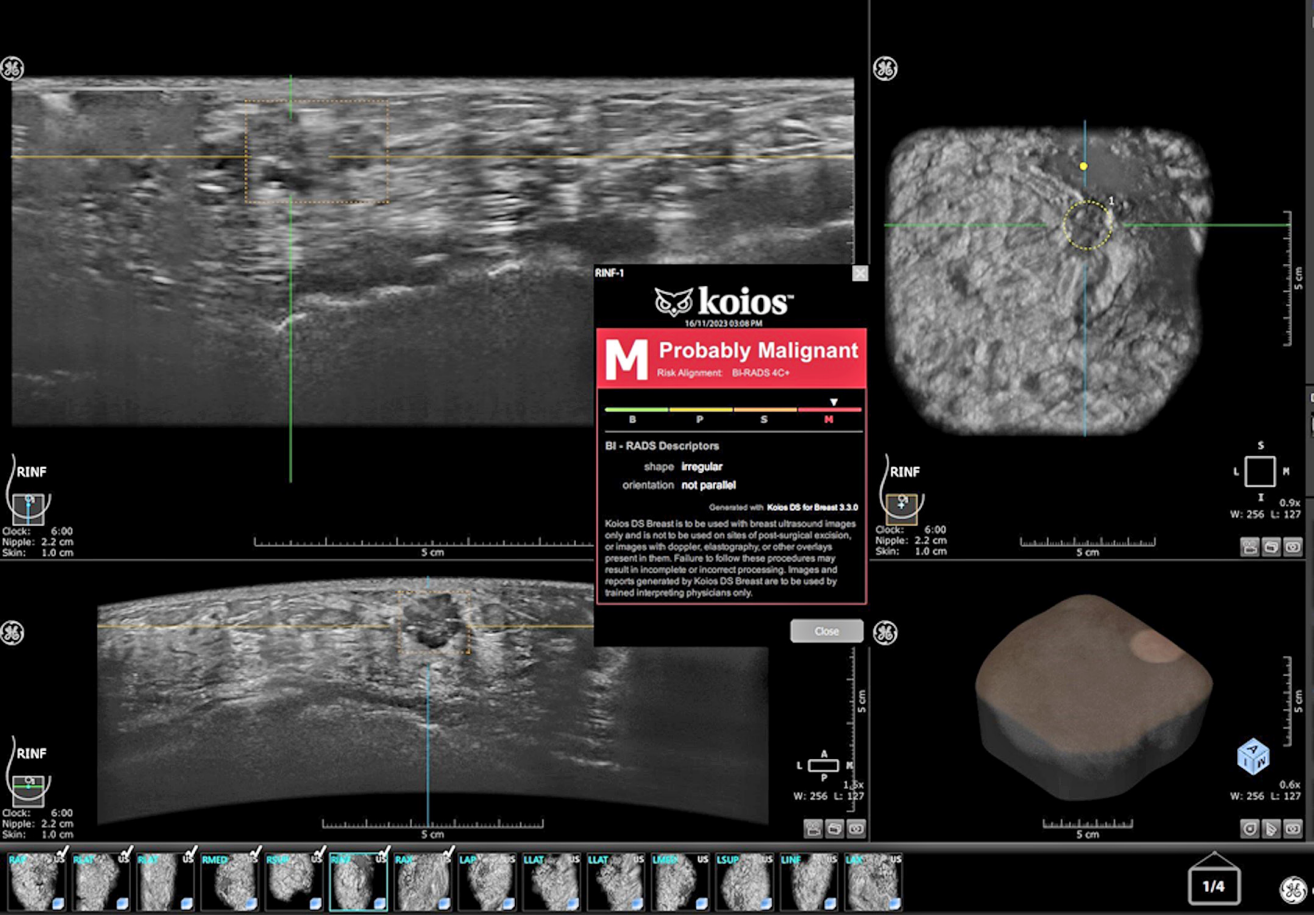
Using machine learning, a high suspicion determination was obtained for the lesion based on breast ultrasound imaging.

Efforts to employ computers in assisting with the identification of breast malignancies have a history of over two decades. Despite substantial interest and investment, traditional computer-aided detection has shown minimal or no substantial enhancement in performance and outcomes. Nevertheless, recent progress in AI, machine learning (ML), and deep learning (DL) is beginning to fulfill the potential for improved performance. Currently, there are many AI applications for breast imaging, but their adoption and utilization vary widely and are generally low (Figure 1). ^4,34-37^ By distinguishing complex patterns in images, AI can transform image interpretation into a more measurable and objective procedure. In a screening environment, AI diminishes the time required for interpretation, lowers the rate of recalls, minimizes the necessity for biopsies, enhances lesion detection, and reduces interval cancers.^38-42^ Radiographic imaging methods enable comprehensive analysis and predictions by efficiently processing significant amounts of data from a variety of sources, including genomics, pathology, and electronic health records.^43^ Additionally, progress in radiogenomics, transcriptomics, and metabolomics enables AI to process intricate multidimensional data, offering unique advantages in assessing breast cancer heterogeneity and broadening the possibilities for a comprehensive exploration of breast cancer’s pathophysiological mechanism in precision medicine.^44-49^

Despite advancements in diagnostic approaches, the existing workflow for breast cancer diagnosis is not infallible. Overdiagnosis, a limited number of professionals (such as breast radiologists and pathologists), natural variations in interpretations, accessibility challenges for examinations, and high costs constitute significant concerns. The role of the radiologist is multifaceted, encompassing diagnostic imaging, biopsy, radiological–pathological correlation, local–systemic staging, treatment decision-making, and follow-up. Breast cancer surveillance involves a longitudinal analysis of tumor changes, with roles in monitoring neoadjuvant chemotherapy response and prognosis. The automatic, measurable, and repeatable nature of AI technology has the potential to precisely monitor lesions. Its anticipated contributions to diagnosis and follow-up include heightened diagnostic accuracy, enhanced performance for general radiologists, and effectiveness in predicting neoadjuvant chemotherapy response and prognosis.^38-42^

Although possessing the potential for significant advancements, the development and utilization of AI encounters a myriad of challenges. The complexities associated with AI arise from various factors such as ethical considerations, technological limitations, legal frameworks, data privacy concerns, and the need for comprehensive testing and validation. Overcoming these hurdles requires a concerted effort from researchers, developers, policymakers, and stakeholders to ensure that AI is harnessed effectively and responsibly in diverse applications.^32,50,51^

## Legal Issues

Over the past 20 years, AI systems have developed quickly, moving from ML to DL and now to transformer models that can use multimodal data as inputs.^52^ Radiology is well positioned to take the lead in the creation and application of AI algorithms as well as handle the associated ethical and legal issues.^53-56^

Numerous challenges are confronted in this process, and more will come up. It is necessary to estimate the present and future barriers to its development and application and take suitable action to overcome them.

Developing robust legal regulations is a critical imperative on a global scale, encompassing a wide array of considerations, from establishing AI teams to engaging end users. A comprehensive approach to forming AI teams involves not only defining tasks and responsibilities but also addressing intricate issues like safeguarding intellectual property rights, ensuring privacy and personal rights, compliance with regulatory guidelines, and clearly delineating the duties, responsibilities, and rights of involved institutions.^57^ This landscape also includes managing risks related to data security, potential concerns with imaging data, and instituting preventive measures against sabotage or manipulation, all while carefully considering accountability in AI-related decision-making processes.^58,59^

Moreover, the ethical dimensions of AI application in health care necessitate an unwavering commitment to fundamental principles. These principles encompass safeguarding human autonomy, promoting well-being, ensuring safety, and advancing the public interest.^60^ Prioritizing transparency, explainability, and understandability in AI systems is essential, fostering responsibility and accountability at every stage. Additionally, there is an urgent need to champion inclusivity and equality, ensuring the accessibility of AI technologies’ benefits to all. Encouraging sensitive and sustainable development practices is crucial to ensure the responsible deployment of AI in health care and beyond. Establishing a robust legal and ethical framework is not just a necessity but a cornerstone for the successful and conscientious integration of AI technologies into diverse fields.^55,61^

In the critical health-care sector, which concerns everyone, the permanent and effective integration of AI demands close monitoring and supervision by AI-literate health-care professionals, at least initially. Evaluating promised achievements, observing real clinical behaviors, and ensuring correct progress in processes are essential steps. Artificial intelligence-based systems should undergo testing in authentic clinical scenarios, with performance assessments preceding integration into clinical practices. Identifying system deficiencies through feedback and making necessary corrections is imperative. Health-care professionals must comprehend the impact of AI applications on clinical practices, continuously monitor them for reliability, and ensure the necessary trust is established before widespread use, ensuring positive impacts on patient safety and treatment efficacy. A broader spectrum of stakeholders, spanning radiologists, patients, health professionals, and various others, should engage in comprehensive assessments, leveraging their in-depth understanding of both AI data processes and tools. Users must possess a nuanced awareness, not just of the strengths but also of the inherent limitations within AI systems.^62,63^

Beneficence, nonmaleficence, justice and fairness, safety, dependability, data security, privacy and confidentiality, bias reduction, openness, explainability, and autonomy are all important factors to take into account when it comes to imaging science. Artificial intelligence is only a tool; humans get to decide how best to use it. Applications of AI can improve clinical and research practices while also fostering more meaningful and in-depth connections between patients and doctors. However, medical imaging norms and recommendations must be carefully considered in light of ethical, legal, and social issues.^64^

Artificial intelligence in breast imaging faces data challenges encompassing size, diversity, quality, and sharing. Size limitations, driven by privacy and technical issues, impact model performance, urging collaboration and data enlargement. However, sharing is hindered by confidentiality and regulatory concerns. Data quality relies on proper labeling, cleaning, and objective processing, with reliable labeling and automation enhancing accuracy.^55,65,66^ Anonymization and security measures are crucial for addressing privacy concerns in data sharing.^56,63,67-73^

The processing of the health data required for the operation of AI systems is possible either with the data owner’s explicit consent or by anonymizing the relevant data. Obtaining the explicit consent of each data owner is very costly regarding time and other aspects.^74,75^ The other option, anonymization, is also a costly process and may lead to a decrease in the system’s performance due to the loss of some information necessary for the AI system to function well. Another effect of the high cost of both options is that health data can only be obtained from organizations that can afford to bear these costs, and therefore the data remains limited. This situation, on the other hand, may have a negative effect in the form of AI systems trained with limited data reaching erroneous conclusions, as well as discrimination as a legal problem against individuals and groups that are not represented in the available data due to sample size disparity.^74,76^

Artificial intelligence grapples with the black box problem and trust issues, requiring model transparency and physician trust. Achieving interpretability demands clear methodologies, transparent models, and radiologists’ confidence. Effective human–machine communication is pivotal for fostering patient trust. Essential strategies to overcome challenges like the black box problem and trust issues in AI involve enhancing model transparency and trust. Establishing guidelines for interpretability, promoting transparent algorithms, and addressing biases is crucial for improving predictability. In recent years, AI has gained prominence in medical imaging, specifically through DL techniques like artificial neural networks. Excelling in tasks such as image preprocessing, registration, detection, and segmentation, DL systems sometimes outperform humans. However, the growing complexity and opacity of DL models pose challenges in result interpretation, hindering the understanding of their advanced prediction mechanisms.^77-80^ Most importantly, the extent and possible consequences of a system’s choices in the context of the application domain are closely linked to the requirement for explainability.^80^ Explainability is also a legal requirement. Especially in terms of liability law, when damage that can be associated with the AI system occurs, to find where the error is and thus decide who will be liable for the damage requires that the conclusions of the system be explainable.^57^

Deep learning systems offer a broad range of applications due to the multiple layers in artificial neural networks, a key component that enables flexible mapping functions between input data and desired outputs. The intricate training phase approximates this mapping, but the complexity often hinders the direct interpretation of results. As AI solutions become more complex with trillion-parameter ranges, human understanding lags behind, necessitating efforts to supervise these systems. Explainability in AI has gained prominence to address this gap, aiming not only for functional benefits but also for enhancing clinical confidence and ensuring compliance with legal and ethical standards. The resurgence of interest in interpretability comes after the pursuit of high performance, especially in critical decision-making scenarios where human experts seek to comprehend and control AI systems.^80^

Model architectures may shape input data, influencing factors like dimensionality, resolution, and layout, leading to transformations such as dimensionality reduction. Hardware constraints, encompassing storage, memory, and computing capabilities, not only limit model choices but also contribute to data transformation. Technical constraints extend to challenges in AI tool integration into clinical practice, including compatibility issues with Picture Archiving Communication Systems (PACS) and Radiology Information System (RIS) systems. Efficient algorithm development faces increased difficulty due to the intricate nature of multivariable data crucial for clinical reasoning. This complexity involves multimodality imaging, multiparametric protocols, clinical knowledge, and past or contralateral examinations. Collaboration among stakeholders, including clinicians, data scientists, researchers, industry representatives, policymakers, and patients, is essential in designing and developing AI systems. This collaborative effort should include sharing development tools and procedures, defining common definitions, and establishing metrics for evaluation and model comparison. While the text highlights challenges in selecting and tuning AI model architectures, considering technical constraints and multivariable data complexity, it emphasizes the need for enhanced collaboration among stakeholders.^81-84^ The involvement of legal experts in these teams is pivotal in navigating the legal landscape, ensuring compliance, and offering valuable insights for risk management. Their presence significantly enhances the efficiency and robustness of the process, preempting and addressing legal challenges proactively.

## Conclusion

Integrating AI in breast imaging holds immense promise for revolutionizing diagnostic and screening processes. Despite the advancements and numerous applications, challenges persist, ranging from data issues to the need for transparency and trust in AI systems. Addressing these challenges is crucial for the responsible and effective deployment of AI in breast imaging. Legal and ethical considerations play a pivotal role, necessitating the development of comprehensive regulations, ethical frameworks, and transparent AI models. The collaboration of legal experts within AI teams is essential to navigate the intricate landscape and ensure compliance. Furthermore, a commitment to ethical principles, including transparency, accountability, and inclusivity, is paramount for the successful and conscientious integration of AI technologies. As AI continues to evolve, careful attention to legal, ethical, and social dimensions will be pivotal in maximizing its benefits while minimizing potential risks in the dynamic landscape of breast imaging.
